# Surface Engineering of Natural Killer Cells with Lipid-Based Antibody Capture Platform for Targeted Chemoimmunotherapy

**DOI:** 10.3390/pharmaceutics17101285

**Published:** 2025-10-01

**Authors:** Su Yeon Lim, Yeongbeom Kim, Hongbin Kim, Seungmin Han, Jina Yun, Hyun-Ouk Kim, Suk-Jin Ha, Sehyun Chae, Young-Wook Won, Kwang Suk Lim

**Affiliations:** 1 Department of Smart Health Science and Technology, Kangwon National University, Chuncheon 24341, Republic of Korea; suyeonlim9846@kangwon.ac.kr (S.Y.L.); youngbum0215@kangwon.ac.kr (Y.K.);; 2 Department of Biomedical Engineering, College of Engineering, University of North Texas, Denton, TX 76203-5017, USA; 3 Division of Hemato-Oncology, Department of Internal Medicine, Soonchunhyang University Bucheon Hospital, Bucheon-si 14584, Republic of Korea; 4 Institute of Fermentation of Brewing, Kangwon National University, Chuncheon 24341, Republic of Korea; 5 Department of Biotechnology and Bioengineering, College of Art, Culture, and Engineering, Kangwon National University, Chuncheon 24341, Republic of Korea

**Keywords:** natural killer cell, surface engineering, antibody capture, immunotherapy, targeted delivery

## Abstract

Next-generation cancer immunotherapy increasingly combines tumor-targeting antibodies or antibody–drug conjugates (ADCs) with immune effector cells to enhance therapeutic precision. However, many existing approaches rely on genetic modification or complex manufacturing, limiting their clinical scalability and rapid deployment. To address this issue, we developed an antibody capture protein (ACP)-based surface engineering platform that enables the rapid, reversible, and non-genetic functionalization of NK cells with therapeutic antibodies or ADCs. This approach uses a DMPE-PEG-lipid conjugate to anchor thiolated protein A (ACP) to the NK cell membrane via hydrophobic insertion, thereby stably and selectively binding to the Fc region of IgG molecules. Using this strategy, we developed ACP-modified NK cells (AC-NKs) that can selectively capture therapeutic antibodies (trastuzumab (TZ), trastuzumab-emtansine (T-DM1), and sacituzumab (SZ)) pre-bound to each target antigen on tumor cells and induce antigen-specific cytotoxic responses. The resulting AC-NKs exhibited enhanced tumor recognition and cytotoxicity against HER2-positive and Trop-2-positive cancer cells in vitro. Compared with conventional combination therapies, AC-NKs enhanced immune activation, as demonstrated by effective delivery of cytotoxic agents, enhanced cancer cell engagement, and upregulation of CD107a expression. Notably, the system supports multiple antigen targeting and tunable antibody loading, enabling adaptation to tumor heterogeneity and resistant phenotypes. This platform might also provide a simple, scalable, and safe method for rapidly developing programmable immune cell therapies without genetic modification. Its versatility supports multi-antigen targeting and broad applicability across NK and T cell therapies, offering a promising path toward personalized, off-the-shelf chemoimmunotherapy.

## 1. Introduction

The integration of chemotherapy and immunotherapy, collectively termed chemoimmunotherapy, has emerged as a transformative approach in cancer treatment, leveraging the cytotoxic effects of chemotherapeutic agents and the tumor-specific targeting capabilities of the immune system [1,2,3]. Among chemoimmunotherapeutic agents, therapeutic antibodies such as trastuzumab and sacituzumab or antibody–drug conjugates (ADCs) such as trastuzumab-emtansine (T-DM1) have demonstrated significant clinical efficacy by selective antigen-directed delivery [4,5,6]. However, the therapeutic potential of ADCs remains limited by critical challenges such as intratumoral antigen heterogeneity, dynamic antigen loss, and the inability to fully activate the host immune system [6,7,8]. These limitations often result in incomplete tumor eradication, the development of resistance, and suboptimal long-term outcomes [9,10,11].

To overcome these obstacles, recent strategies have focused on combining ADCs with immune effector cells, such as natural killer (NK) cells, to enhance the therapeutic synergy between direct cytotoxicity and immune-mediated tumor elimination [12,13,14,15]. NK cells possess intrinsic tumor-killing capabilities and can mediate antibody-dependent cellular cytotoxicity (ADCC) in the presence of therapeutic antibodies [12,14,16]. Among the various NK cell platforms, the NK-92 cell line is a well-established, clinically approved “off-the-shelf” therapeutic recognized for its consistent expansion, potent cytotoxic activity, and lack of inhibitory killer immunoglobulin-like receptors (KIRs), which minimizes the risk of graft-versus-host disease [17,18]. NK-92 cells are deficient in FcγRIII (CD16), which impairs IgG-binding and limits antibody- or ADC-driven antitumor activity [15,19].

We developed a lipid-based antibody-capturing protein (ACP) platform that rapidly and non-genetically transforms natural killer (NK) cells into programmable antibody-guided immune effectors. This process relies on the spontaneous insertion of a hydrophobic lipid–polymer conjugate, DMPE-PEG-maleimide, into the NK cell membrane, allowing for covalent anchoring of thiolated Protein A (ACP) via maleimide–thiol coupling [13,14,16]. Once immobilized on the cell surface, ACP selectively binds to the Fc region of therapeutic IgG antibodies or antibody–drug conjugates (ADCs), equipping NK cells with antibody-directed tumor-targeting ability. This strategy enables the modular, reversible, and efficient surface functionalization of immune cells without compromising membrane integrity or cellular viability. Unlike traditional genetic engineering methods, which require an extended processing time and pose risks such as insertional mutagenesis, our approach offers a simple and scalable alternative for functionalizing immune effector cells.

To evaluate the performance of our system, we conjugated ACP-modified NK cells (AC-NKs) with clinically relevant therapeutic agents, including trastuzumab (TZ), trastuzumab-emtansine (T-DM1), and sacituzumab (SZ). These AC-NKs effectively recognized and eliminated HER2-positive and Trop-2-positive cancer cells through the combined action of the NK cells and the surface-bound antibodies or ADCs. Compared to the conventional co-treatment of NK cells with antibodies, AC-NKs demonstrated enhanced tumor targeting, improved the membrane localization of therapeutic antibodies, and upregulated the expression of CD107a, indicating active immune degranulation. Furthermore, the ACP-based anchoring system enabled the flexible, sequential, or simultaneous binding of multiple antibodies, allowing for customizable and multi-antigen targeting. This tunable surface engineering platform has the potential to support personalized immunotherapy by adapting antibody loading levels to match specific therapeutic needs.

Unlike previous one-step methods that directly anchor ADCs on immune cells, our ACP system introduces a modular protein-based binding interface, which enhances stability and adaptability while supporting broader clinical applications [12]. Herein, we established a proof-of-concept demonstrating that ACP stably and reversibly delivers therapeutic antibodies to NK cells, enhancing target binding and cytotoxicity in vitro without genome editing. These in vitro data support the feasibility of a modular approach compatible with the rapid and scalable production of off-the-shelf immune cell products. These findings support the ability of ACP-mediated surface engineering to unlock the combined efficacy of current ADC and NK cell therapies. By reversibly and antigen-independently conjugating therapeutic antibodies to immune cells, this strategy could advance chemoimmunotherapy for solid tumors.

## 2. Materials and Methods

### 2.1. Materials

All therapeutic antibodies included trastuzumab (TZ), sacituzumab (SZ), and trastuzumab emtansine (T-DM1) (Selleckchem, Houston, TX, USA). DMPE-PEG-maleimide (Molecular weight = 2 kDa) and Protein A-thiol were obtained from Nanocs (New York, NY, USA) and Protein Mods (Waunakee, WI, USA), respectively. Proteins were fluorescently labeled with NHS-fluorescein or NHS-rhodamine (Thermo Fisher Scientific, Waltham, MA, USA). Cell culture used Dulbecco’s modified Eagle’s medium (DMEM) and RPMI 1640 (Luscience, Hanam, Republic of Korea) supplemented with recombinant human interleukin-2 (IL-2; Lonza, Walkersville, MD, USA). Viability and apoptosis were assessed with Cell Counting Kit-8 (CCK-8, Dojindo Molecular Technologies, Kumamoto, Japan) and the PE Annexin V/7-AAD kit (BioLegend, San Diego, CA, USA), respectively. FITC-conjugated anti-human CD107a and APC-conjugated anti-human CD56 and CD244 were obtained from BD biosciences (San Jose, CA, USA) and Biolegend (San Diego, CA, USA), respectively. For the Western blot assay, anti-rabbit IgG, anti-6 × His tag, and HRP-conjugated secondary antibodies were sourced from Cell Signaling Technology (Danvers, MA, USA). Enzyme-linked immunosorbent assays (ELISA) employed the ClinMax™ Human Granzyme B kit (ACRO Biosystems, Seoul, Republic of Korea) and the Human Perforin kit (Invitrogen, Carlsbad, CA, USA). Unless otherwise specified, all reagents were analytical grade. Confocal laser scanning microscopy (CLSM; Carl Zeiss, Oberkochen, Germany) and flow cytometry analyses (FACS Calibur and FACSymphony, Becton Dickinson, Franklin Lakes, NJ, USA) were performed at Kangwon National University (Chuncheon, Republic of Korea).

### 2.2. Production of a PEG-DMPE Conjugated with Antibody-Capturing Protein

To generate antibody-capturing protein (ACP) conjugated with PEG-DMPE, DMPE-PEG-maleimide was dissolved in distilled water and incubated at 60 °C until completely dissolved. Thiol-modified protein A was dissolved separately in PBS (phosphate-buffered saline, Welgene, Gyeongsan, Republic of Korea). The two components were mixed at a molar ratio of 10:1 (DMPE-PEG-maleimide: Protein A) to promote conjugation via maleimide–thiol bonding. The reaction was maintained at 4 °C with gentle agitation overnight. After thiol–maleimide coupling, unreacted excess DMPE-PEG-maleimide was eliminated from Protein A-PEG-DMPE through dialysis against cold 1× PBS twice. The concentration of the resulting DMPE-PEG-protein A conjugate was quantified using a DC protein assay kit (Bio-Rad, Hercules, CA, USA).

### 2.3. Cell Culture

SK-BR-3 and Calu-3 (HER2-positive) and MDA-MB-231 (HER2-negative) were obtained from the Korea Cell Line Bank (KCLB, Seoul, Republic of Korea) and maintained per the supplier’s protocol. SK-BR-3 and MDA-MB-231 cells were grown in RPMI 1640 containing 10% FBS (MilliporeSigma, St. Louis, MO, USA) and antibiotics (100 U/mL penicillin, 100 µg/mL streptomycin). Calu-3 cells were cultured in Dulbecco’s Modified Eagle Medium (DMEM) with the same concentrations of supplements. All adherent cell lines were cultured at 37 °C in a humidified atmosphere containing 5% CO_2_ and passaged every 2–3 days when approximately 80% confluence was reached. The NK-92 human natural killer cell line was purchased from the American Type Culture Collection Center (ATCC, Manassas, VA, USA) and maintained according to ATCC guidelines. Cells were maintained in α-MEM containing 20% FBS, 1% penicillin–streptomycin, 4 mM folic acid, 20 mM inositol, 0.02 mM 2-mercaptoethanol, and 100 U/mL recombinant human IL-2. Suspension cultures of NK-92 cells were cultured under the same temperature and carbon dioxide conditions as the cancer cell lines and passaged every 2 days to maintain optimal cell density.

### 2.4. Optimization of Surface Modification Condition for Production of Antibody-Capturing NK Cell (AC-NK) Using ACP-PEG-DMPE

Rhodamine was conjugated with an antibody-capturing protein to optimize the modification of NK cells using ACP-PEG-DMPE. Rhodamine-conjugated ACP-PEG-DMPE was mixed with NK cells (1 × 10^6^ cells) at varying concentrations of ACP-PEG-DMPE for 15 min at room temperature (RT). We also prepared AC-NKs using 40 μg of ACP-PEG-DMPE for 10 to 40 min at RT.

### 2.5. Evaluation of Cytotoxicity and Growth in ACP-PEG-DMPE Treated NK Cells

To assess the effect of ACP-PEG-DMPE on NK cell activity during the generation of antibody-capturing NK (AC-NK) cells, we assessed both the cytotoxicity of C3-PEG-DMPE on NK-92 cells and the proliferative capacity of AC-NKs. NK-92 cells were cultured with 0 (Control group), 10, 20, or 40 μg of C3-PEG-DMPE at 37 °C in a 5% CO_2_ atmosphere for 24 h to evaluate the cytotoxic effects. The AC-NKs were prepared by mixing NK cells with various amounts of ACP-PEG-DMPE for 15 min under RT. The AC-NKs were then maintained under the same culture conditions for 48 h. The viability and proliferation of NK cells after modification were measured using the CCK-8 kit following the manufacturer’s protocol. The absorbance at 450 nm was detected using a microplate reader (Biotek, Winooski, VT, USA).

### 2.6. Confirmation of the Antibody Capturing of AC-NKs and the Formation of AC-NKs

We synthesized rhodamine-tagged ACP-PEG-DMPE to examine its surface localization on NK cells. Rhodamine-labeled ACP-PEG-DMPE was used to treat NK cells for 15 min at RT. The AC-NKs were mixed with FITC-labeled Trastuzumab at RT for 15 min and then washed with 1× PBS. After detachment, all samples were fixed in 4% PFA (paraformaldehyde) prepared in 1× PBS. They were stored at 4 °C until FACS and confocal laser scanning microscope (CLSM) analyses. The fluorescence of NK cells with rhodamine-labeled ACP-PEG-DMPE and FITC-labeled Trastuzumab was analyzed by flow cytometry and CLSM at the core facility of Kangwon National University (Chuncheon, Republic of Korea).

### 2.7. Characterization of AC-NKs with Trastuzumab

The availability of NK cell receptors after the AC-NKs were bound with trastuzumab (TZ) was analyzed using APC-labeled CD56 and CD244(2B4). Each antibody was applied onto AC-NKs bound with TZ, following the manufacturer’s recommendations. The presence of CD56 and CD244 on AC-NKs bound with TZ was determined using FPLC (Fast Protein Liquid Chromatography) and analyzed using FlowJo software v10.10.0.

### 2.8. Verification of Specific Binding of AC-NKs to Trastuzumab That Recognized HER2 Positive Cancer Cells

All cancer cells, including Calu-3, SK-BR-3, and MDA-MB-231, were labeled with Cell Tracker Blue CMAC for evaluation of the HER2-positive cancer cell targeting ability of the AC-NKs to bind with TZ. They were plated at 5 × 10^4^ cells per well 24 h before treatment. The cancer cells were seeded in wells at a density of 5 × 10^4^ cells/well 24 h prior to treatment. AC-NKs were prepared using rhodamine-conjugated ACP-PEG-DMPE. All cancer cells were incubated with 5 μg of FITC-labeled trastuzumab (TZ) for 30 min and then washed with MEM α (minimum essential medium alpha) twice. After washing, each cancer cell was co-incubated with AC-NKs for 30 min. The location of FITC-TZ and AC-NKs was observed using CLSM. All CLSM images were analyzed using ImageJ software v2.16.0.

To quantify the number of remaining AC-NKs after co-incubation, all cancer cells and NK cells were stained using CMAC (blue) and CMTPX (Red), respectively. Then, 5 μg of trastuzumab was used to treat all cancer cells for 30 min. After incubation, the cells were washed with MEM α twice. Each cancer cell was mixed with NK or AC-NKs at an *E:T* (*effector-to-target*) of 10:1 for 30 min. Unbound NK cells were washed with 1× PBS, and the collected remaining cells were analyzed using flow cytometry.

### 2.9. In Vitro Anti-Cancer Efficacy of AC-NKs via Capturing Trastuzumab (TZ)

To determine the anti-cancer availability of AC-NKs with antibodies, trastuzumab (TZ) was used to treat cancer cells, and then AC-NKs were treated. HER2-positive cancer cells (SK-BR-3, Calu-3, and BT-474), and HER2-negative cancer cells (MDA-MB-231), were labeled with Cell Tracker Blue CMAC. All cancer cells were seeded at 5 × 10^4^ cells/well in a 24-well plate and incubated for 24 h at 37 °C and 5% CO_2_. Then, they were incubated with 5 μg of TZ for 1 h at 37 °C and 5% CO_2_ and washed with MEM α. They were co-incubated with NK cells and AC-NKs at an *E:T ratio* of 10:1 for 30 min. After co-incubation, unbound NK cells and AC-NKs were washed with MEM α in all treatment groups, and the remaining cancer cells bound with effect cells were further incubated for 24 h. To analyze cancer cell death, an annexin V assay kit was processed according to the manufacturer’s recommended protocol and analyzed using flow cytometry at the core facility of Kangwon National University (Chuncheon, Republic of Korea).

### 2.10. The Mechanism of AC-NKs’ Anti-Cancer Effect Through Capturing Trastuzumab (TZ)

The synergetic anti-cancer effect of the AC-NKs and TZ was quantified by the expression level of CD107a in HER2-positive cancer cells, such as SK-BR-3, Calu-3, and BT-474, and a HER2-negative cancer cell, including MDA-MB-231. Cancer cells were plated at 5 × 10^4^ cells per well in a 48-well plate and cultured at 37 °C and 5% CO_2_ for 24 h. Then, 5 μg of TZ was used to treat all cancer cells for 1 h at 37 °C and 5% CO_2_, and these were then washed with MEM α. They were co-cultured with NK cells or AC-NKs at an *E:T ratio* of 1:1 for 30 min. After incubation, unbound NK and AC-NKs were washed with MEM α in all treatment groups. FITC-conjugated anti-CD107a was used to treat the remaining cells. After 2 h, all cells in each well were collected and stained with APC-labeled CD56 antibody for NK cell identification. Fluorescence was measured by flow cytometry at the Kangwon National University core facility (Chuncheon, Republic of Korea).

### 2.11. In Vitro Determination of Anti-Cancer Efficacy of AC-NKs Through Antibody Capturing

To assess the synergetic anti-cancer effect of AC-NKs via antibody capturing, we treated trastuzumab-emtansine (T-DM1) and sacituzumab (SZ) into HER2-positive cancer cells, such as BT-474 and SK-BR-3, and TROP-2-positive cancer cells, including MCF-7 and SK-BR-3, respectively. All cancer cells were stained with Cell Tracker Blue CMAC, seeded at 5 × 10^4^ cells per well in 24-well plates, and then incubated for 24 h under cell culture conditions (37 °C and 5% CO_2_). Then, 5 μg of T-DM1 and SZ were used to treat HER2-positive cancer cells and TROP-2-positive cancer cells, respectively. They were then co-cultured with NK cells and AC-NKs at an *E:T ratio* of 10:1 for 30 min. After co-incubation, unbound NK cells and AC-NKs were washed with MEM α in all treatment groups, and then the remaining cells were further cultured for 24 h. To analyze cancer cell death, an annexin V assay kit was processed according to the manufacturer’s recommended protocol and analyzed using flow cytometry at the core facility of Kangwon National University (Chuncheon, Republic of Korea).

### 2.12. Statistical Analysis

All data are shown as mean ± SD (standard deviation). Comparisons used Student’s *t*-test. * *p* < 0.05, ** *p* < 0.01, and ^#^ *p* < 0.001 values were considered statistically significant.

## 3. Results

### 3.1. Schematic Illustration for Targeted Cancer Immunotherapy Using Antibody-Capturing Protein

**Figure 1:**
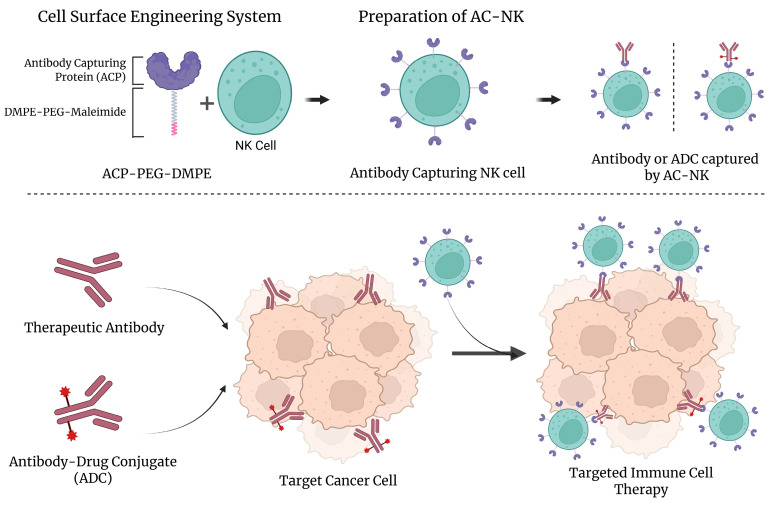
Schematic illustration of antibody capturing NK cells (AC-NK) for targeted immune cell therapy. The schematic illustration shows a cell surface engineering approach that enhances the anti-tumor efficacy of NK cells by enabling antibody capture on the cancer cell. NK cells are functionalized with ACP-PEG-DMPE, a lipid-polymer conjugate that incorporates an antibody-capturing protein (ACP) into the NK cell membrane. Through this modification, AC-NK cells are capable of binding therapeutic antibodies or antibody–drug conjugates (ADCs) directed against antigens on cancer cells. These engineered NK cells engage tumor cells via the captured antibodies, leading to immune synapse formation. Upon target recognition, AC-NK cells release cytotoxic effectors, inducing apoptosis in the cancer cells.

The antibody-capturing protein (APC) used for antibody capturing on the NK cells surface consists of protein A with thiol (Figure 1). Protein A is bound to immunoglobulin and is widely used for antibody capture in applications such as purification and detection [20,21,22]. To generate a cell surface engineering system, protein A needs to be conjugated with PEG-DMPE. The recombinant protein A with thiol was purchased from Protein Mods. This protein has a small number of sulfhydryl groups that can be used to react with gold or maleimide. The antibody-capturing protein linked with PEG-DMPE (ACPD) was generated according to a previous report [13,14].

### 3.2. Optimization and Characterization of Antibody-Capturing NK Cells (AC-NKs) Using ACP-PEG-DMPE with NK Cells

**Figure 2:**
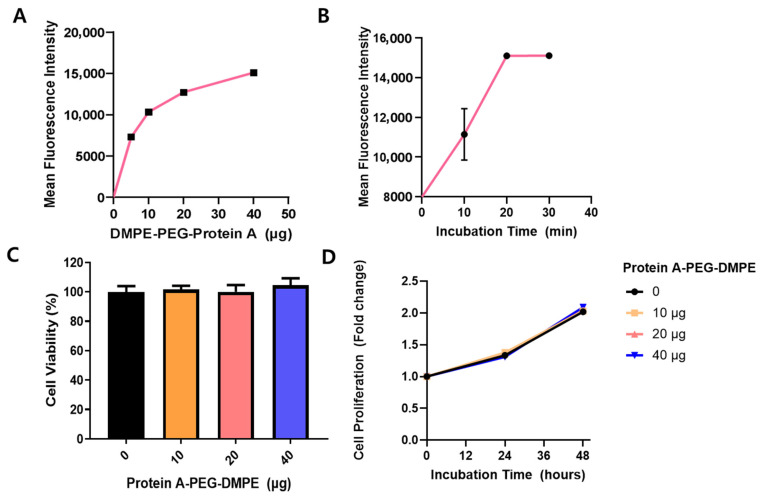
Optimization and characterization of antibody-capturing NK cell (AC-NK) using ACP-PEG-DMPE with NK cells. AC-NK cells were produced by incubating NK cells (1 × 10^6^ cells) with various concentrations and durations of rhodamine-conjugated ACP-PEG-DMPE. (**A**) NK cells were treated with increasing amounts of rhodamine-ACP-PEG-DMPE to determine the optimal concentration for surface modification. (**B**) NK cells were incubated with 40 μg of rhodamine-ACP-PEG-DMPE for different time periods to evaluate time-dependent incorporation. (**C**) Comparison of NK cell viability between unmodified NK cells (0 μg) and AC-NK cells generated with 10, 20, and 40 μg of ACP-PEG-DMPE. (**D**) Comparison of NK cell proliferation between unmodified NK cells (0 μg) and AC-NK cells treated with 10, 20, and 40 μg of ACP-PEG-DMPE. ACP: Antibody capturing protein (protein A); AC-NK: antibody capturing NK cell.

To generate antibody-capturing NK cells (AC-NKs) using ACP-PEG-DMPE, NK cells were exposed to various concentrations of ACP-PEG-DMPE for 15 min. As shown in Figure 2A, rhodamine-labeled Protein A-PEG-DMPE was observed on the NK cells in a dose-dependent manner (5 min: 7331.00 ± 131.56; 10 min: 10,333.67 ± 102.96; 20 min: 12,723.67 ± 108.10; 40 min: 15,106.00 ± 262.04; mean ± SD). To evaluate the incorporation of rhodamine–Protein A-PEG-DMPE onto the NK cells (AC-NKs), flow cytometry analysis was conducted. As depicted in Figure 2B, the fluorescence intensity of rhodamine–Protein A increased proportionally with its concentration, confirming cell surface modification (10 min: 11,141.00 ± 1059.68; 20 min: 15,094.00 ± 52.43; 30 min: 15,105.67 ± 112.27). ACP-PEG-DMPE effectively transformed the whole NK cell population within 20 min. Across various amounts, ACP-PEG-DMPE surface modification did not affect NK cell viability or proliferation (Figure 2C,D). To confirm the cytotoxicity, NK cells were treated with ACP-PEG-DMPE at concentrations of 0, 10, 20, and 40 μg and incubated for 24 h. The cytotoxicity in each NK-cell treatment group was assessed using the CCK-8 assay. NK-cell viability remained approximately 100% in all groups, indicating no cytotoxicity of ACP-PEG-DMPE at the tested concentration (Figure 2C). The modified cells were then cultured for 48 h, and their growth was compared to that of unmodified NK-92 cells. As shown in Figure 2D, the AC-NKs exhibited a comparable proliferation rate to unmodified NK-92 cells across all treatment concentrations. Cell growth was unchanged across groups, indicating that ACP-PEG-DMPE at these doses does not impair NK-cell viability or proliferation over 48 h. Therefore, NK cells were treated with 40 μg of ACP-PEG-DMPE to generate AC-NKs.

### 3.3. Confirming Antibody-Capturing Availability of AC-NKs

**Figure 3:**
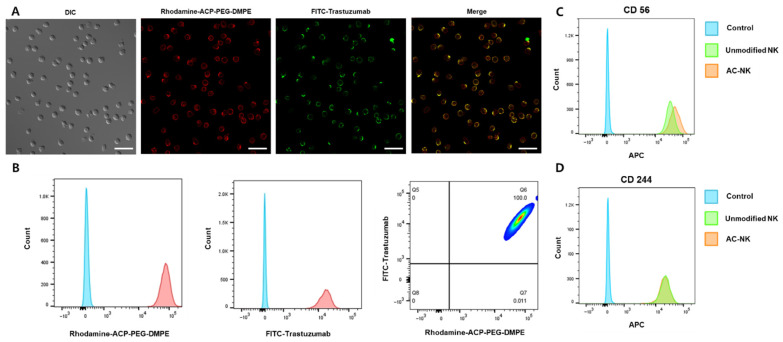
Confirmation of antibody capturing and phenotypic characterization of antibody-capturing NK cell (AC-NK). (**A**) Surface modification and antibody capture on NK cells using ACP-PEG-DMPE. NK cells (1 × 10^6^ cells) were incubated with 40 μg of rhodamine-conjugated ACP-PEG-DMPE for 15 min, followed by incubation with 20 μg of trastuzumab for 15 min. Fluorescence intensity on AC-NK was observed using confocal laser scanning microscopy (CLSM). (**B**) Quantification of dual-positive NK cells with rhodamine conjugated ACP-PEG-DMPE and FITC-trastuzumab by flow cytometry. Blue: Control; Red: AC-NK. (**C**) Expression level of CD 56 on the surface of AC-NK. (**D**) Expression level of CD244 on the surface of AC-NK. Unmodified NK cells and AC-NK cells were stained with APC-conjugated CD56 and CD244 antibodies, respectively. Fluorescence of each antibody was analyzed by flow cytometry to confirm the preservation of receptor expression following surface engineering. Quantification of dual-positive NK cells with rhodamine conjugated ACP-PEG-DMPE and FITC-trastuzumab by flow cytometry. DIC: Differential interference contrast microscopy. ACP: Antibody capturing protein (protein A); ACP-PEG-DMPE: ACP conjugated with PEG-DMPE; AC-NK: antibody capturing NK cell. Scale bars: 10 μm.

To verify ACP-mediated AC-NK/antibody complex formation at the NK-cell surface, we assessed the surface localization of ACP-PEG-DMPE and the antibody on NK cells. As shown in Figure 3A, surface localization of rhodamine-tagged ACP-PEG-DMPE and FITC-trastuzumab (TZ) on NK cells was detected. FITC-TZ colocalized with rhodamine-labeled ACP-PEG-DMPE, and ~100% of cells were double-positive for rhodamine and FITC (Figure 3B). These findings indicate that the ACP moiety in ACP-PEG-DMPE mediates efficient antibody capture on the surface of the NK cell. TZ was present on the surface of AC-NK/TZ cells, and surface expression of NK markers CD56 and CD244 was maintained. These data indicate that TZ remains on the NK-cell surface with no evidence of internalization. Furthermore, TZ loading did not reduce receptor accessibility on engineered NK cells, indicating their intrinsic cytolytic function is preserved (Figure 3C,D). Because NK-92 cells are deficient in Fc receptors CD16, CD32, and CD64, they are unable to trigger internalization or ADCC (antibody-dependent cellular cytotoxicity) [16]. ACP-PEG-DMPE-based surface engineering had little impact on NK cell metabolism or viability.

### 3.4. Specific Binding of AC-NKs to Trastuzumab Targeting HER2-Positive Cancer Cells

AC-NKs were co-incubated with trastuzumab (TZ)-treated HER2-positive (SK-BR-3, Calu-3) and HER2-negative (MDA-MB-231) cancer cells to evaluate their binding ability. At 30 min, AC-NKs (red) were observed forming clusters with the HER2-positive cancer cells, where FITC-TZ (green) was clearly localized on the cancer cell membrane, confirming TZ binding. In contrast, the MDA-MB-231 cells (HER2 negative) showed minimal FITC-TZ signals and limited association with the AC-NKs. After 12 h, the AC-NKs remained attached to the HER2-positive cancer cells, and strong intracellular green fluorescence was detected, indicating TZ internalization and sustained NK cell interaction. However, in the HER2-negative cells, only a small number of AC-NKs were detected, and no significant fluorescence signal was observed. These results demonstrate that AC-NKs selectively recognize and engage with HER2-positive cancer cells via the action of trastuzumab, maintaining stable interactions over extended incubation periods, whereas interactions with HER2-negative cells remain minimal.

**Figure 4:**
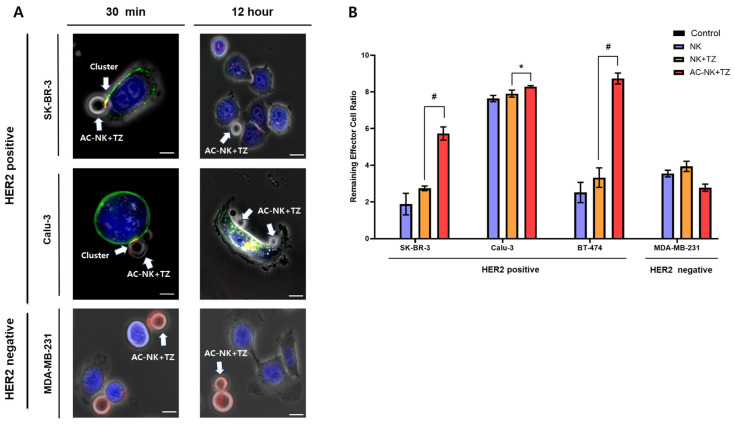
Verification of the specific binding of AC-NK to trastuzumab, which recognizes HER2-positive cancer cells. (**A**) To evaluate the targeting ability of AC-NK toward trastuzumab that recognizes HER2-positive cancer cells, the AC-NK was treated with HER2-positive cancer cells and HER2-negative cancer cells. Each cancer cell was labeled with CMAC (blue). AC-NK was prepared using rhodamine-labeled ACP-PEG-DMPE. FITC-conjugated TZ was incubated with cancer cells for 30 min and then washed with MEM α twice. After washing, cancer cells were co-incubated with AC-NK cells for 30 min or 12 h. Unbound NK cells were removed by washing, and the remaining cells were visualized using CLSM. The white arrow indicated AC-NK cells successfully targeting HER2-positive cancer cells by specifically binding to TZ. (**B**) The remaining NK cell was also quantified using FACS analysis. The 5 μg of trastuzumab was added to all cancer cells for 30 min and then washed with MEM α twice. Each cancer cell was co-incubated with an NK cell and an AC-NK cell at an *effector-to-target (E:T) ratio* of 10:1 for 30 min. Data are presented as mean ± SD (*n* = 3). Statistical significance was determined by Student’s *t*-test (* *p* < 0.05, ^#^ *p* < 0.001). ACP: Antibody capturing protein; AC-NK: antibody capturing NK cell. Scale bar: 10 µm.

To assess the trastuzumab (TZ) specific binding, cancer cells were pretreated with TZ, cultured with AC-NKs, and the number of NK cells remaining with HER2-positive versus HER2-negative targets was quantified. Unmodified NK cells or AC-NKs were co-cultured with HER2-positive targets (SK-BR-3, Calu-3, BT-474) or HER2-negative MDA-MB-231 cells at an *E:T ratio* of 10:1 for 30 min. After co-incubation, unbound NK cells were removed by washing, and the retained cells were counted by flow cytometry. As shown in Figure 4B, the remaining effector cell counts (mean ± SD) for unmodified NK vs. AC-NKs were SK-BR-3, 2.74 ± 0.10 vs. 5.73 ± 0.29; Calu-3, 7.91 ± 0.16 vs. 8.28 ± 0.05; BT-474, 3.33 ± 0.44 vs. 8.73 ± 0.24. In MDA-MB-231 cells, the residual NK cell counts of untransformed NK cells and untransformed NK cell + TZ did not show a significant difference. However, the residual NK cell counts of AC-NKs were lower than those of untransformed NK cells, which appears to be because AC-NKs specifically target TZ. These results revealed that AC-NKs specifically recognize and bind to TZ on HER2-positive cancer cells.

### 3.5. In Vitro Anti-Cancer Effect of AC-NKs with TZ (Trastuzumab)

**Figure 5:**
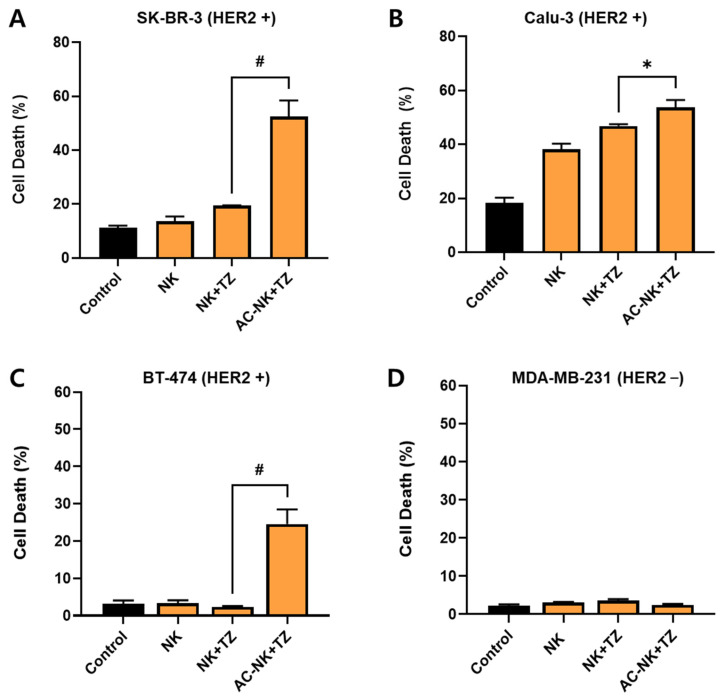
In vitro anti-cancer efficacy of AC-NK with Trastuzumab. To determine the anti-cancer effect of AC-NK with trastuzumab (TZ), all cancer cells were labeled with cell tracker blue CMAC. (**A**) SK-BR-3; (**B**) Calu-3; (**C**) BT-474; (**D**) MDA-MB-231. They were pre-treated with 5 μg of TZ for 1 h, followed by co-incubation with either NK cells or antibody-capturing NK (AC-NK) cells at an *effector-to-target (E:T) ratio* of 10:1 for 30 min. After removal of unbound NK cells, they were further cultured for 24 h and stained with Annexin V to evaluate cancer cell death. Flow cytometry analysis revealed that AC-NK with TZ induced significantly higher levels of apoptosis in HER2-positive cancer cells compared to NK with TZ. No significant anti-cancer efficacy was observed in HER2-negative MDA-MB-231 cells, demonstrating the HER2-specific cytolytic activity of AC-NK via captured TZ. Data are presented as mean ± SD (*n* = 3). Statistical significance was determined by Student’s *t*-test (* *p* < 0.05, ^#^ *p* < 0.001). ACP: Antibody capturing protein (protein A); AC-NK: antibody capturing NK cell.

The anti-cancer activity of AC-NKs with TZ (trastuzumab) was confirmed in HER2-positive lines (SK-BR-3, BT-474, Calu-3) and in HER2-negative cells (MDA-MB-231). To compare AC-NKs + TZ with NK + TZ, cancer cells were pretreated with TZ and then co-cultured with AC-NKs or NKs for 30 min at an *E:T ratio* of 10:1. After co-incubation, the cells were washed with PBS and cultured for an additional 24 h, and cancer cell death was measured using an apoptosis assay kit. As shown in Figure 5, AC-NKs with TZ enhanced the death of HER2-positive cells than other treatment groups: SK-BR-3 (52.40 ± 4.70), BT-474 (24.57 ± 3.30), and Calu-3 (53.73 ± 2.65). In particular, the anti-cancer effect was more prominent in SK-BR-3 cells than in Calu-3 cells, which is likely due to the higher expression level of HER2 in SK-BR-3 cells [23,24]. The anti-cancer activity of AC-NK containing TZ in HER2-positive cells is attributed to specific binding to tumor-bound TZ via AC-mediated antibody capture. In contrast, MDA-MB-231 (HER2-negative cancer) did not show significant cell death, demonstrating the treatment’s specificity.

### 3.6. The Mechanism of Action of the Anti-Cancer Effect of AC-NKs with Trastuzumab (TZ)

**Figure 6:**
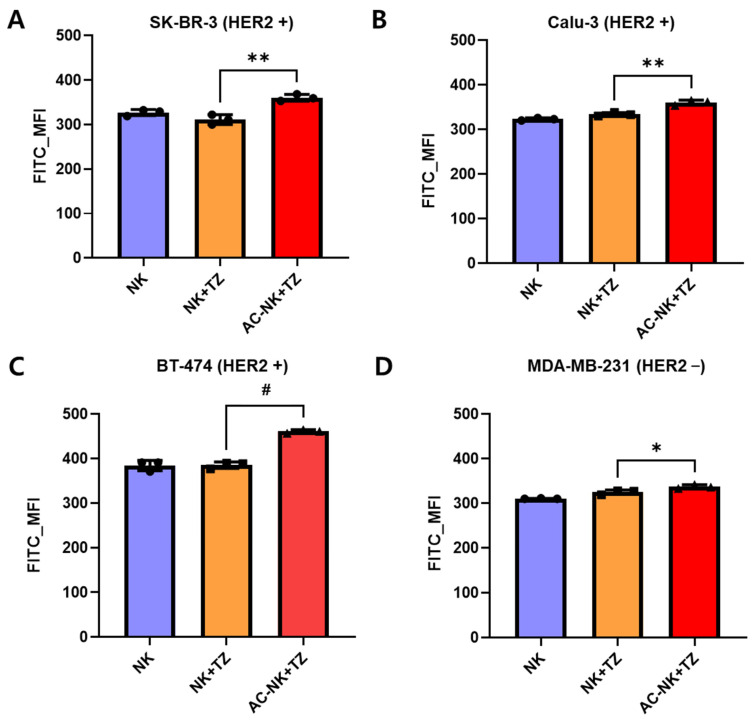
The mechanism of action of the AC-NK cell with Trastuzumab. To evaluate the cytotoxicity potential of AC-NK cells with trastuzumab (TZ), the anti-107a antibody was measured in NK cells, NK + TZ cotreatment, and AC-NK + TZ cotreatment. (**A**) SK-BR-3; (**B**) Calu-3; (**C**) BT-474; (**D**) MDA-MB-231. All cancer cells were pre-treated with 5 μg of TZ for 1 h. They were incubated with either unmodified NK cells or AC-NK at an *effector to target (E:T) ratio* of 1:1 for 30 min. After removal of unbound effector cells, remaining NK-bound cancer cells were incubated with FITC-conjugated anti-CD107a antibody for 2 h and stained with APC-labeled anti-CD56 to identify NK cells. CD107a expression level was significantly elevated in AC-NK cells upon interaction with HER2-potisive targets, particularly SK-BR-3 and BT-474, while no increase was observed with HER2-negative MDA-MB-231. Data are presented as mean ± SD (*n* = 3). Statistical significance was determined by Student’s *t*-test (* *p* < 0.05, ** *p* < 0.01, ^#^ *p* < 0.001). ACP: Antibody capturing protein (protein A); AC-NK: antibody capturing NK cell.

To clarify the mechanism of AC-NK/TZ activity, CD107 release was quantified in treated HER2-positive (SK-BR-3, BT-474, Calu-3) and HER2-negative (MDA-MB-231) cells. As shown in Figure 6, AC-NKs with TZ elicited significantly higher CD107a levels than other treatments in co-cultures with HER2-positive targets (SK-BR-3, BT-474, Calu-3). Relative to Calu-3, the greater cytotoxicity observed in SK-BR-3 and BT-474 is consistent with their higher HER2 expression, which increases trastuzumab-binding efficiency. In HER2-negative MDA-MB-231 cells, CD107a did not increase, indicating that TZ-dependent activation of AC-NKs is restricted to HER2-positive targets. These data suggest that AC-NK with TZ exerts antitumor activity by enhancing NK cell cytotoxicity upon recognition of HER2-expressing targets. ACP-PEG-DMPE–mediated TZ capture confers antigen specificity and potentiates NK effector functions, yielding efficient tumor cell elimination.

### 3.7. In Vitro Anti-Cancer Efficacy of AC-NKs with Antibody–Drug Conjugate or Antibody

**Figure 7:**
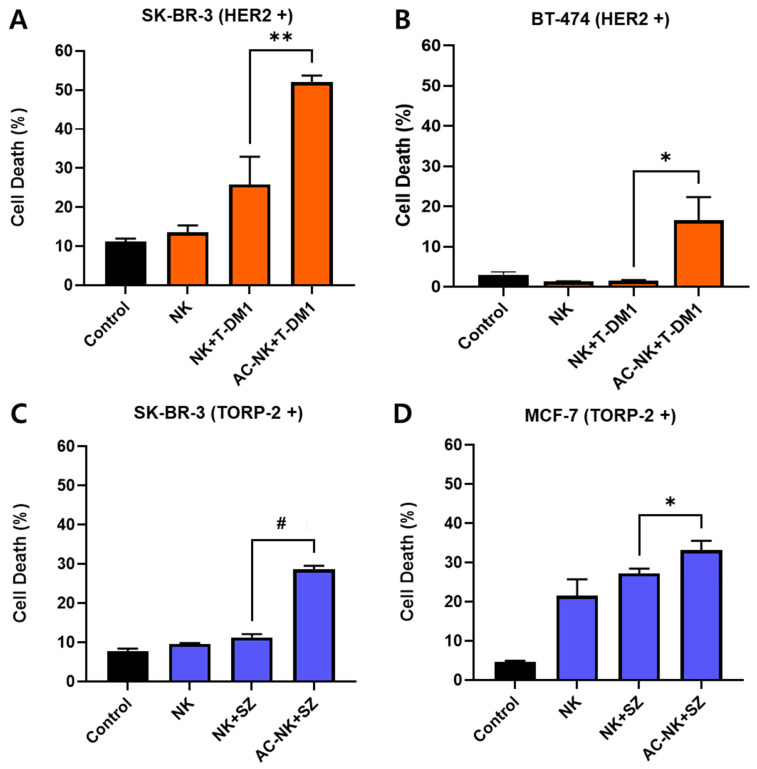
In vitro anti-cancer efficacy of AC-NK as a surface engineering platform system. To confirm the anti-cancer effect of AC-NK with therapeutic antibody or antibody-drug conjugate (ADC), HER2-positive (SK-BR-3 and BT-474) and Trop-2-positive (SK-BR-3 and MCF-7) cancer cells were pre-treated with 5 μg of trastuzumab-emtansine (T-DM1) or sacituzumab (SZ), respectively. They were followed by co-incubation with either unmodified NK cells or AC-NK cells at an *effector-to-target (E:T) ratio* of 10:1 for 30 min. After removal of unbound NK cells, the remaining cancer cells were cultured for 24 h and analyzed for cell death using an annexin V apoptosis assay. (**A**,**B**) AC-NK with T-DM1 significantly enhanced cell death in HER2-positive (**A**) SK-BR and (**B**) BT-474 cells compared to other groups. (**C**,**D**) AC-NK with SZ induced significantly higher cancer cell death in Trop-2 positive (**C**) SK-BR-3 and (**D**) MCF-7 cells compared to other groups. Data are presented as mean ± SD (*n* = 3). Statistical significance was determined by Student’s *t*-test (* *p* < 0.05, ** *p* < 0.01, ^#^ *p* < 0.001). ACP: Antibody capturing protein (protein A); AC-NK/antibody capturing NK cell.

To evaluate the antibody-capturing ability of AC-NKs, trastuzumab-emtansine (T-DM1) and sacituzumab (SZ) were used to treat HER2-positive cancer cells, such as SK-BR-3 and BT-474, and Trop-2 positive cancer cells, such as SK-BR-3 and MCF-7, respectively. To evaluate the therapeutic effect of AC-NK with T-DM1 or SZ, cancer cell lines were pretreated with T-DM1 or SZ and then co-cultured with the corresponding effector groups for 30 min at an *E:T ratio* of 10:1. After incubation, cells were washed with PBS and further cultured for 24 h, after which cancer cell death was quantified using a cell death assay kit. As shown in Figure 7A,B, AC-NKs with T-DM1 significantly enhanced cancer cell death in HER2-postive cancer cells such as SK-BR-3 (52.03 ± 1.65) and BT-474 (16.50 ± 5.85) compared to the other treatment groups. AC-NKs with SZ also improved cancer cell death in Trop-2-positive cancer cells such as SK-BR-3 (29.60 ± 0.55) and MCF-7 (33.20 ± 1.00) compared to the other treatment groups (Figure 7C,D). The observed anti-cancer efficacy of AC-NKs with T-DM1 or SZ in each targeted cancer cell was attributed to its specific binding to T-DM1 or SZ on HER2-positive cancer cells or Trop-2 positive cancer cells, respectively. These results demonstrate that AC-NKs specifically bind to the antibody via AC-mediated antibody capturing on the NK cells.

## 4. Discussion

Cell surface engineering is increasingly being utilized to enhance the efficacy of immune cells and impart novel functions. However, conventional chemical and physical modification strategies can disrupt cell membrane structure and inhibit cellular activity, limiting their translational utility [12,16,25]. To address these limitations, lipid–polymer structures, in which hydrophobic lipid anchors are inserted into the cell membrane and hydrophilic polymer spacers provide functional moieties to the extracellular environment, have been widely reported [26,27]. These systems enhance biocompatibility, reduce nonspecific interactions, and enable modular, reproducible assembly suitable for therapeutic applications. Among the lipid anchors, 1,2-dimyristoyl-sn-glycero-3-phosphoethanolamine (DMPE) was selected due to its strong hydrophobic anchorage to cell membranes and compatibility with immune cells [14,16]. The phosphor ethanolamine head group of DMPE enables efficient conjugation of functional proteins, supporting modular and reproducible assembly [28,29]. Polyethylene glycol (PEG) was conjugated to DMPE to form a lipid–polymer composite that increases solubility and enables modular functionalization. Furthermore, adjustment of PEG chain length establishes a defined nanoscale separation from the plasma membrane, thereby reducing geometric interference and improving accessibility for additional functional moieties.

In this study, we selected PEG with a molecular weight of 2kDa to prepare PEG-DMPE, according to a previous study showing balanced membrane insertion efficiency while minimizing steric hindrance [12,30]. By introducing a thiol-modified protein A (antibody capture protein, ACP) onto this PEG-DMPE scaffold, we produced an AC platform that enables noncovalent capture of therapeutic antibodies and antibody–drug conjugates on the immune cell surface. Furthermore, we selected protein A, rather than protein G, as the Fc-binding module in this study. A protein A binds with high affinity to human IgG1, IgG2, and IgG4, but with weaker binding to IgG3, whereas protein G binds to all four human IgG subclasses, including IgG3 [31,32]. Since the therapeutic antibodies evaluated here (trastuzumab, T-DM1, and sacituzumab) are human IgG1, both antibody binding proteins are compatible. However, protein A avoids albumin binding, which is present in some protein G variants, and the construct used in this study provides a multivalent Fc-binding domain that facilitates high-density IgG capture in mobile membranes [33,34]. These characteristics motivated the use of Protein A in this AC platform.

These results indicate that AC-NKs preserve the antibody-mediated tumor-targeting function and enhance antigen-specific recognition by NK cells. Unlike the co-treatment of unmodified NK cells with therapeutic antibodies, which showed a low anti-cancer effect in both HER2-positive and Trop-2 positive cells, the co-treatment of AC-NKs with a therapeutic antibody such as trastuzumab (TZ), trastuzumab-emtansine (T-DM1), and Sacituzumab (SZ) improved their anti-cancer efficacy in HER2-positive and Trop-2 positive cells. The lipid-based anchoring method could allow therapeutic antibodies to move dynamically along the NK cell membrane after being captured by an antibody-capturing protein on the surface of NK cells. Compared with unmodified NK cells treated with TZ, T-DM1, or SZ, AC-NK exhibited significantly stronger target cell recognition and cytotoxicity as evidenced by increased CD107a expression. These data are consistent with previous reports demonstrating the tumor-selective activity of ADCs immobilized directly or via an intermediate scaffold. Similarly, a previous study using T-DM1-modified NK cells demonstrated no cytotoxicity against HER2-negative cells [12,13,14].

The ACP-mediated surface modification platform offers reversibility, tunability, and adaptability, allowing the temporal control of immune cell activation. Because the antibody capture protein is anchored via noncovalent lipid insertion, antibody loading and unloading can be dynamically regulated under physiological conditions. By adjusting the concentration and incubation time of ACP or antibodies, the degree of surface functionalization can be precisely tuned to optimize therapeutic efficacy and minimize off-target effects. This flexibility supports the sequential or simultaneous targeting of multiple tumor antigens and enables rapid retargeting in response to antigen changes, which are important for overcoming tumor heterogeneity and immune evasion. In addition, the ACP platform is cost-effective, safe, and compatible with existing cell manufacturing workflows. It requires only a simple, mild culture step without complex infrastructure or extended cell culture, significantly reducing the time and cost associated with production. By avoiding permanent genetic modifications, the risks of insertional mutagenesis or unintended genomic alterations are avoided, providing a safer, more accessible, and clinically scalable approach for developing next-generation immune cell therapies

The ACP-based surface engineering system offers a promising solution to the major limitations of current cancer immunotherapy, particularly for solid tumors characterized by antigenic heterogeneity and immunosuppressive microenvironments, by enabling rapid, reversible, and multi-antigen functionalization without genetic manipulation. The efficacy of AC-NK, demonstrated in both HER2-positive and Trop-2-positive cancer models, demonstrates its potential as a modular, off-the-shelf immunotherapy platform. By simply exchanging therapeutic antibodies or ADCs, the system can be readily tailored to target a wide range of malignancies, including those with dynamic or mixed antigen expression. The scalability, safety, and flexibility of the ACP system position it as a next-generation immunotherapy approach with strong translational potential. Future studies will focus on extending this strategy to primary NK cells and T cells, assessing in vivo persistence and biodistribution, and applying it to combination therapies including immune checkpoint blockade or CAR engineering. Overall, our findings demonstrate that ACP-mediated surface engineering represents a versatile and clinically viable platform for personalized antibody-directed cell-based immunotherapy.

## 5. Conclusions

We reported a one-step, non-genetic method that uses a lipid-anchored antibody-capture protein to rapidly and reversibly load therapeutic antibodies or antibody–drug conjugates onto NK cell surfaces. By immobilizing thiolated protein A via DMPE-PEG-mediated membrane insertion, we generated antibody-captured NK cells (AC-NKs) that retained cytotoxicity while acquiring antigen-specific targeting function. This modular approach preserved NK cell viability and enhanced tumor selectivity without the genetic or chemical modification of the therapeutics. When co-administered with clinically approved drugs such as trastuzumab (TZ), trastuzumab-emtansine (T-DM1), or sacituzumab (SZ), AC-NKs exhibited significantly enhanced antitumor efficacy in HER2- and Trop-2-positive tumor models. This platform enables multi-antigen targeting and tunable antibody loading, providing the flexibility to overcome tumor heterogeneity and immune evasion mechanisms. These results demonstrate that this ACP system can serve as a universal interface to rapidly transform immune cells into programmable tumor-targeting therapeutics. Importantly, this approach is broadly applicable to other immune effector cells, including T cells, dendritic cells, and CAR-engineered cells, and is compatible with a variety of therapeutic antibodies and ADCs currently in development. Within the limitations of this in vitro study, ACP-based surface engineering enables NK cells to bind therapeutic antibodies and ADCs in an antigen-dependent manner. Translational studies require the in vivo evaluation of persistence, biodistribution, safety, and the impact of endogenous IgG and other serum proteins that can occupy protein A and induce background or sink effects. Strategies able to mitigate these effects (e.g., ex vivo preloading, Fc binding affinity modulation, and ACP density control) are therefore needed. Therefore, this study presents the use of AC-NKs as a modular platform with translational potential that requires further preclinical evaluation rather than a clinically validated tool.

## Data Availability

The datasets presented in this article are not readily available because the data are part of an ongoing study. Requests to access the datasets should be directed to kslim@kangwon.ac.kr.
